# The m.3290T > C variant might be a protective factor against the pathogenic m.3243 A > G variant: a case study

**DOI:** 10.1186/s13023-025-03774-5

**Published:** 2025-06-13

**Authors:** Ning Zhang, Zhikang Zhang, Ying Zhang, Xun Su, Yuzhou Gao, Jing Yang, Weiwei Zou, Yunxia Cao, Dongmei Ji

**Affiliations:** 1 Department of Obstetrics and Gynecology, the First Affiliated Hospital of Anhui Medical University, No 218 Jixi Road, Hefei, 230022 Anhui China; 2 NHC Key Laboratory of Study on Abnormal Gametes and Reproductive Tract, Anhui Medical University, No. 81 Meishan Road, Hefei, 230032 Anhui China; 3 Key Laboratory of Population Health Across Life Cycle (Anhui Medical University), Ministry of Education of the People’s Republic of China, No. 81 Meishan Road, Hefei, 230032 Anhui China

**Keywords:** Mitochondrial DNA, Mitochondrial disease, m.3290T > C, m.3243A > G, MELAS

## Abstract

The mitochondrial m.3243 A > G variant is a prevalent mitochondrial disease mutation that causes multisystem maternal inheritance disorders. While clinical severity typically correlates with mutation load, symptom manifestation may be influenced by other variants and environmental factors. Notably, the m.3290T > C variant has been hypothesized as a potential protective variant for m.3243 A > G pathogenicity, though clinical evidence remains limited. Here we reported a six-generation Chinese pedigree carrying both m.3243 A > G and homoplasmic m.3290T > C variants. Clinical and genetic analyses revealed that carriers with extremely high m.3243 A > G heteroplasmy (> 95%) exhibited severe symptoms, whereas those with moderate or high levels showed limited or no clinical symptoms. Our findings provide novel evidence for the protective role of m.3290T > C in mitigating m.3243 A > G pathogenicity, highlighting its potential clinical significance.

To the editor,

Mitochondrial diseases are severe maternally inherited disorders, with an incidence rate of approximately 1:4,300 [1]. Mitochondrial DNA (mtDNA) variants are the primary cause of mitochondrial diseases. The pathogenic m.3243 A > G variant is one of the most prevalent variants causing mitochondrial disease and was first reported in 1990 [2]. It impacts the structural stability, methylation, acylation, or codon recognition of tRNALeu^(UUR)^, thus impairing mitochondrial functions, disturbing multiple organs and systems, and leading to primary mitochondrial disorders, including mitochondrial encephalopathy, lactic acidosis, and stroke-like episodes (MELAS) [3] and maternally inherited diabetes and deafness (MIDD) [4]. The mutation level of the m.3243 A > G variant is typically heteroplasmic, and it exhibits a lower symptomatic threshold. With the increase in the variant level, its clinical symptoms become more serious [5]. In addition to heteroplasmy, other factors, including age, mtDNA copies, nuclear genetics, other mtDNA variants and the environment, may also affect the phenotypes of m.3243 A > G variant carriers [6].

In 1995, Hammans et al. [7] reported a three-generation m.3243 A > G pedigree carrying a homoplasmic m.3290T > C variant, where some family members harbored high heteroplasmy of the m.3243 A > G variant but lacked typical symptoms. These results suggest that m.3290T > C could protect against dysfunction induced by the m.3243 A > G variant; however, there are no similar reported pedigrees subsequently. Here, we first report a six-generation Chinese pedigree carrying both m.3243 A > G and homoplasmic m.3290T > C variants, providing additional clinical evidence for the protective function of m.3290T > C.

The proband VI-1 was a 2-year-old boy. He was admitted to a hospital for dyspnea and pneumonia at 20 days of age, and a ventilator was used to assist breathing. Blood examinations revealed a slight increase in lactate levels, and cytomegalovirus infection was detected. MRI of the brain revealed unremarkable. Whole-exome sequencing of the blood samples derived from the proband and his parents identified no relevant pathogenic variant. However, NGS-based mtDNA sequencing confirmed the presence of m.3243 A > G, with a 98.43% variant load in the proband. He was further diagnosed with MELAS syndrome and died of heart failure at 2.5 years of age. His mother sought genetic counseling for this reason and hereby came to our hospital. After informed consent was obtained, the medical history of the pedigree was obtained. Blood, buccal mucosa, nail, and hair samples from pedigree members were collected for mtDNA genetic analyses. In brief, DNA extracted from samples was subjected to whole genome amplification. Subsequently, mtDNA-specific amplification and Sanger sequencing combined with droplet digital polymerase chain reaction (ddPCR) were conducted. Details on the genetic analysis can be found in our previous article [8]. The clinical characteristics, detection methods, and m.3243 A > G variant loads are presented in Table 1. The pedigree was characterized mainly by cardiomyopathy, and all the family members carried both the m.3243 A > G variant and the homoplasmic m.3290T > C variant (Fig. 1). The proband’s mother V-1, aged 30 years, had an 88.87% variant level of m.3243 A > G and was reportedly normal. The proband’s uncle V-2, aged 27 years, was reportedly normal, with a 66.00% variant level of m.3243 A > G. IV-2 had an 86.9% variant level of m.3243 A > G but reported no symptoms. IV-3, aged 44 years with an 80.20% variant level of m.3243 A > G, was diagnosed with tachycardia and liver cirrhosis and returned to normal after treatment. Currently, the patient has hypertension and is taking antihypertensive drugs. IV-6 and IV-7 were normal. IV-11 died at 30 years of age with heart failure, and the details were unavailable. Additional genetic and nongenetic factors for death cannot be excluded because of the consanguineous marriage of the patients. III-3 carried an 86.9% variant level of m.3243 A > G and presented only an increased heart rate occasionally. The m.3243 A > G variant was not detected in blood samples of III-4, IV-4, or V-3.

**Table 1:** m.3243 A > G variant load and main medical history of the carriers

Subject No.	Sex	Age (years)^a^	Affected^a^	Medical history^a^	m.3243 A > G variant load^a, b,c^
II-1	Female	70	Yes	History of cardiomyopathy; died at the age of 70; the cause of death was unknown	N.D.
II-2	Female	N.D.	N.D.	N.D.	N.D.
III-1	Female	30	Yes	History of cardiomyopathy; died at the age of 30; the cause of death was unknown	N.D.
III-2	Female	3	Yes	History of cardiomyopathy; died at the age of 3; the cause of death was unknown	N.D.
III-3	Male	N.D.	No	Increased heart rate occasionally	B:84.45%
III-4	Male	N.D.	No	Reported normal	B:0.00%
III-5	Female	N.D.	Yes	History of cardiomyopathy	N.D.
III-6	Female	N.D.	No	Reported normal	N.D.
IV-1	Female	3	Yes	History of cardiomyopathy; died at the age of 3; the cause of death was unknown	N.D.
IV-2	Female	47	No	Reported normal	B:86.90%; H:77.89%
IV-3	Male	46	Yes	Diagnosed with hypertension, tachycardia, and liver cirrhosis, returned to normal after treatment	B:80.20%
IV-4	Female	N.D.	No	Reported normal	B:0.00%; BM:0.00%
IV-5	Male	N.D.	No	Reported normal	N.D.
IV-6	Female	N.D.	No	Reported normal	B:30.75%; BM: 31.30%
IV-7	Female	N.D.	No	Reported normal	B:29.70%; BM: 28.80%
IV-8	Female	N.D.	N.D.	N.D.	N.D.
IV-9	Male	N.D.	N.D.	N.D.	N.D.
IV-10	Male	N.D.	No	Reported normal	N.D.
IV-11	Male	30	Yes	History of cardiomyopathy; died at the age of 30; the cause of death was heart failure	N.D.
V-1	Female	27	No	Reported normal	B:88.87%; N: 88.95%; H: 88.87%
V-2	Male	24	No	Reported normal	B:66.00%; H:76.35%
V-3	Female	N.D.	No	Reported normal	B:0.00%; BM:0.00%
V-4	Male	N.D.	No	Reported normal	N.D.
V-5	Male	N.D.	Yes	History of cardiomyopathy	N.D.
V-6	Male	N.D.	No	Reported normal	N.D.
VI-1^d^	Male	2.5	Yes	Diagnosed with MELAS; age of onset was 20 days with dyspnea and pneumonia as first symptoms; died at the age of 2.5; the cause of death was heart failure; slightly increased blood lactate; Brain MRI was unremarkable	B:98.43%

^a^ Not Determined: N.D

^b^ Blood: B; buccal mucosa: BM; Nail: N; Hair: H

^c^ For VI-1, variant load was detected via NGS; for others, variant load was detected via Sanger sequencing combined with ddPCR

^d^ Proband

**Fig. 1:**
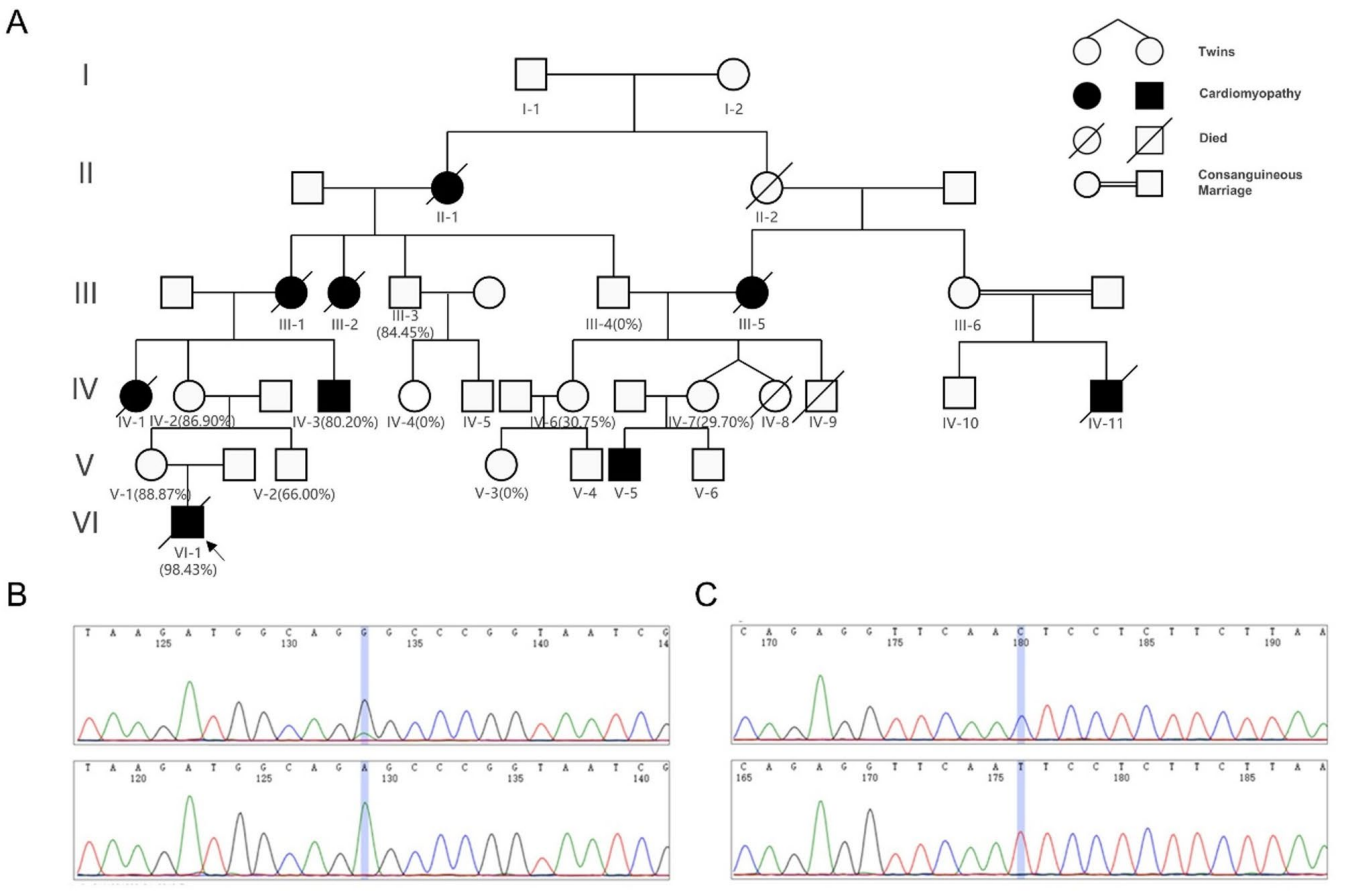
Pedigrees carrying both m.3243 A > G and homoplasmic m.3290T > C variants. (**A**) Pedigree diagram of the family. The numbers in parentheses are the heteroplasmy levels of m.3243 A > G in the blood. The black symbols indicate patients with cardiomyopathy. The arrow represents the proband. (**B**) Heteroplasmy of m.3243 A > G variant in V-1’s blood (upper) and control sample (lower) via Sanger sequencing. (**C**) Homoplasmy of m.3290T > C variant in V-1’s blood (upper) and control sample (lower) via Sanger sequencing

There are few studies on mitochondrial DNA variant interactions at present. Kozin et al. reported that m.12308 A > G and m.10398 A > G variants interacted synergistically to increase the risk of breast cancer [9]. Other studies have indicated that mtDNA variant interactions might influence Alzheimer’s disease and multiple sclerosis risk [10, 11]. The m.3290T > C variant was first found in a hypotonic infant, in cases of sudden infant death syndrome and focal segmental glomerulosclerosis, and in pedigrees of essential hypertension and was previously considered pathogenic [12–15]. However, it is now widely acknowledged as a benign variant and a protective factor against m.3243 A > G [16]. In this pedigree, some carriers (III-3, IV-2, V-1, and V-2) with high levels of m.3243 A > G and homoplasmic m.3290T > C did not exhibit clinical symptoms. Only the proband with an extremely high level of m.3243 A > G (98.43%) presented with MELAS. Given the attenuation effect of the heteroplasmy level of m.3243 A > G in blood, this phenomenon is quite unusual [17]. The same phenomenon was also observed in the study of Hammans et al. [7], and carriers with high levels of m.3243 A > G (> 80% in blood) and homoplasmic m.3290T > C presented only limb weakness or were reported to be normal. In addition, they reported that uncloned cell lines derived from the patient displayed a distinct genetic pattern, with the heteroplasmy level of m.3243 A > G remaining stable after six passages. These results further validated the protective role of m.3290T > C.

However, the specific mechanisms underlying the protective effect of the m.3290T > C variant are still unknown. We speculate that this protective effect may be related to the function of tRNALeu ^(UUR)^. The m.3243 A > G variant destabilizes the structure of tRNALeu^(UUR)^, causing low structural stability and impaired mitochondrial oxidative phosphorylation. In contrast, the structural alterations resulting from the m.3290T > C variant unexpectedly serve a compensatory role to maintain tRNALeu^(UUR)^ function and repair mitochondrial protein synthesis [6, 18]. In addition, environmental influences and nuclear genetics might be potential confounding factors. The penetrance and clinical phenotype of multiple mitochondrial diseases have been demonstrated to be associated with environmental factors including smoking, alcohol, and drugs [19, 20]. Nuclear factors are closely associated with the clinical phenotypes of the m.3243 A > G variant [17]. For example, increased expression of the nuclear gene *MNRR1* could rescue the cellular defects caused by the m.3243 A > G variant [21]. However, the influence of environmental factors and nuclear genetics on m.3290T > C remains unclear. Future studies are needed to elucidate the compensatory mechanism of the m.3290T > C variant against the m.3243A>G variant. In vitro models, such as hybrid cell lines harboring m.3243 A > G alone versus m.3243 A > G and m.3290T > C, could reveal differences in mitochondrial complex activity. CRISPR/Cas9-mediated introduction of m.3290T > C into m.3243 A > G carrier-derived fibroblasts could further validate its functional impact. In addition, in vivo validation using mouse models engineered to carry both variants could be used to assess tissue-specific protection and phenotypic amelioration.

There are also other reported cases with both m.3243 A > G and m.3290T > C variants. Loh et al. [22] reported a 13-year-old girl diagnosed with MELAS, and she carried the m.3243 A > G variant with a heteroplasmy level of 50% and the m.3290T > C variant (mutation level unknown). In addition, at the age of 10, she underwent posterior spinal instrumented fusion surgery due to adolescent idiopathic scoliosis. Therefore, it remains unclear whether her condition was associated with anesthetic agents, surgery, or other causes [23]. Her sister was also diagnosed with MELAS and carried homoplasmic m.3243 A > G and m.3290T > C variants. In addition, she also had severe scoliosis and was treated with surgery. This finding is consistent with our findings, indicating that patients carrying a notably high level of the m.3243 A > G variant and the homoplasmic m.3290T > C variant may still develop conditions.

In conclusion, our study provides new clinical evidence for the protective role of the m.3290T > C variant against the pathogenic m.3243 A > G variant, which highlights the need to consider protective variants in genetic counseling and interventions. A better understanding of the potential biological mechanisms underlying the protective effect of the m.3290T > C variant could favor the development of therapies for m.3243 A > G variant diseases. In future research, we will further explore the potential mechanism and its influence on the genetic patterns of the m.3243 A > G variant.

## Data Availability

The data supporting the conclusions of this article are included within the article. Owing to ethical restrictions, other data are available upon request.
