# Correction: The m.3290T > C variant might be a protective factor against the pathogenic m.3243 a > g variant: a case study

**DOI:** 10.1186/s13023-025-03872-4

**Published:** 2025-07-01

**Authors:** Ning Zhang, Zhikang Zhang, Ying Zhang, Xun Su, Yuzhou Gao, Jing Yang, Weiwei Zou, Yunxia Cao, Dongmei Ji

**Affiliations:** 1 Department of Obstetrics and Gynecology, the First Affiliated Hospital of Anhui Medical University, No 218 Jixi Road, Hefei, 230022 Anhui China; 2 NHC Key Laboratory of Study on Abnormal Gametes and Reproductive Tract, Anhui Medical University, No. 81 Meishan Road, Hefei, 230032 Anhui China; 3 Key Laboratory of Population Health Across Life Cycle (Anhui Medical University), Ministry of Education of the People’s Republic of China, No. 81 Meishan Road, Hefei, 230032 Anhui China

**Correction: Orphanet J Rare Dis 20**,** 306 (2025)**

10.1186/s13023-025-03774-5

Following publication of the original article [1], the authors reported an error in the authorship order.

The authorship order was published as:

Ning Zhang^1,2,3†,^ Zhikang Zhang^1,2,3†,^ Ying Zhang^1,2,3^, Xun Su^1,2,3,^ Yuzhou Gao^1,2,3,^ Jing Yang^1,2,3,^ Weiwei Zou^1,2,3^, Dongmei Ji^1,2,3*^ and Yunxia Cao^1,2,3*^.

The authorship order has been corrected to:

Ning Zhang^1,2,3†,^ Zhikang Zhang^1,2,3†,^ Ying Zhang^1,2,3^, Xun Su^1,2,3,^ Yuzhou Gao^1,2,3,^ Jing Yang^1,2,3,^ Weiwei Zou^1,2,3^, Yunxia Cao^1,2,3*^and Dongmei Ji^1,2,3*^.

The original article [1] has been updated.
